# Lung-Centered Open Heart Surgery: A Call for a Paradigm Change

**DOI:** 10.3389/fcvm.2016.00012

**Published:** 2016-05-12

**Authors:** Edward Gologorsky, Angela Gologorsky, Tomas Antonio Salerno

**Affiliations:** ^1^Anesthesiology, Allegheny General Hospital, Pittsburgh, PA, USA; ^2^Anesthesiology, Private Practice, Miami Beach, FL, USA; ^3^Cardiovascular Surgery, University of Miami Miller School of Medicine, Miami, FL, USA

**Keywords:** cardiopulmonary bypass, postoperative complications, pulmonary dysfunction, capnography, ischemia–reperfusion injury

“The time has come,” in the famous words of Lewis Carroll, “to talk of many things,” and we would like to talk about many things that lead to severe pulmonary dysfunction after cardiac surgery. The term is broad indeed and is usually taken to represent a wide gamut ranging from well-compensated abnormalities of respiratory mechanics to symptomatic hypoxemia secondary to ventilation/perfusion mismatching to more significant prolonged ventilator dependency to dreaded “respiratory cripple.” Overwhelming financial and societal costs aside, prolonged ventilator dependency carries the staggering in-house mortality in excess of 40% (1). The incidence and severity was reported to vary widely between centers, partially because of disparate definitions, but significantly because of poorly defined “hospital quality” characteristics (2, 3). We agree with those who look for the causes outside the various demographic characteristics, but rather focus on performance, perioperative surgical and anesthetic techniques, experience and expertise of surgical perioperative care team (4, 5). Let us concentrate on what we actually do in the operating room and see what can be improved.

Let us follow, in broad strokes, what happens to the lungs in the course of a traditional intraoperative care of a patient presenting for an open heart surgery. To start, the lungs are ventilated with F_i_O_2_ = 1.0, large tidal volumes, and zero end-expiratory pressure until the extracorporeal circulation is established. At that moment the mechanical ventilation is completely suspended, with resultant profound iatrogenic atelectasis. Concurrently, as venous return is diverted into cardiopulmonary bypass (CPB) circuit, pulmonary arterial flow ceases, rendering lungs dependent on bronchial arterial flow. The latter, normally approximately 10% of the nutrient flow, is highly variable during the bypass period as it is determined by systemic pressures and flows. Thus, ischemic and atelectatic organ, subjected to oxygen toxicity and ventilator-induced injury during pre-bypass period, is now exposed to CPB-induced systemic inflammatory response (SIRS), greatly potentiated by sequestration of activated polymorphonuclear leukocytes in pulmonary capillaries. After completion of CPB, the lungs are again subjected to repeated stretch trauma of “bag squeezing” recruitment maneuvers, and face additional injury due to reperfusion, potentiated by reactive oxygen species in hyperoxic (F_i_O_2_ = 1.0) milieu. Additionally, the right ventricular performance may be impaired in the early post-bypass period due to cardioplegia-induced edema and swelling, regional tissue electrolyte, metabolic and temperature heterogeneity, and ischemia–reperfusion injury (silent ischemia is common and is frequently undetected in a quiescent myocardium). Note that protamine administration typically takes place during the early reperfusion period of both lungs and myocardium; we believe that hemodynamic manifestations of ARDS-like pulmonary injury and RV dysfunction greatly potentiate the so-called “protamine reaction.”

“Vision,” Jonathan Swift remarked, “is the art of seeing things invisible.” Akin to an iceberg, lung injury during cardiac surgery is a process clinically significant in only a minority of patients. But we know that it takes place in all patients, and though frequently undetected and unsuspected, may be devastating when manifested. Patient descriptors usually associated with prolonged ventilator dependency after cardiac surgery, such as age, emphysema, heart failure, renal failure, prolonged CPB, complex procedures, and significant transfusion requirements, can all be understood as pathologic preconditions that, to the degree that they exhaust the adaptive compensatory mechanisms, greatly potentiate the noxious effects of pulmonary volutrauma, atelectrauma, biotrauma, and ischemia–reperfusion injury in hyperoxic and inflammatory milieu, allowing the ARDS-like syndrome to be manifested earlier and more severe.

Insanity is said to be “doing the same thing over and over again, and expecting different results.” So what could be done differently for patients at risk for postoperative pulmonary dysfunction? We advocate lung-centered paradigm of intraoperative management of a cardiac surgical patient. Changes are needed both in anesthetic management and in surgical gestalt. In addition to numerous improvements in CPB circuit design and perfusion techniques geared toward lessening the inflammatory response (5–7), we specifically advocate adoption of protective pulmonary ventilation and concurrent continuous pulmonary perfusion and ventilation throughout the CPB period.

Despite being introduced into general clinical practice more than a decade ago, protective pulmonary ventilation in cardiac surgery is still not routine (8). This concept includes ventilating with lower tidal volumes, and advocates titrated positive end-expiratory pressure (PEEP) and recruitment maneuvers to maintain patency of alveoli and lower F_i_O_2_ to avoid oxygen toxicity and absorption atelectasis while maintaining acceptable oxyhemoglobin saturations (9, 10). Once initiated with induction of general anesthesia, it should be continued throughout the entire perioperative period including intensive care unit (ICU). We suspect that its implementation in cardiac surgery has been hampered by the apprehension of its futility in the face of induced prolonged iatrogenic atelectasis and pulmonary ischemia, concerns regarding the effects of PEEP on RV function, and traditional reliance on high FiO_2_ to mask the resultant hypoxemia. However, maintaining protective pulmonary ventilation during the bypass period would address all of these concerns. In fact, some preliminary data suggest improved patients’ outcomes with mitigated ventilator-induced injury and atelectrauma in both off-pump on on-pump cardiac surgery (11, 12).

So why did some earlier studies of pulmonary ventilation during bypass not find a significant effect on postoperative pulmonary function? (13). We believe that the absence of simultaneous pulmonary perfusion in these studies provides the answer (14). Ventilating ischemic alveoli is not likely to be of any lasting benefit, just as perfusing atelectatic lungs may exacerbate the alveolar edema and would be of questionable value. Despite some enthusiastic reports describing the benefits of isolated pulmonary perfusion (15–17), we insist that to be clinically significant, pulmonary perfusion should be matched with pulmonary ventilation. While the details and the specifics of the technique of pulmonary perfusion during bypass period still await further studies, in our practice, we use a 3-mm cannula connected to the port of the aortic cannula to perfuse the pulmonary artery with arterial blood. Simultaneous capnography provides the key to this technique, as observation of end-tidal CO_2_ waveform provides a continuous, reliable, and reproducible evidence of alveolar perfusion and ventilation (18).

It is highly probable that simultaneous pulmonary perfusion and pulmonary protective ventilation may not only mitigate the noxious effect of ventilator trauma, atelectasis, and ischemia–reperfusion injury, but may also prevent bacterial translocation and diminish SIRS, as reported by Richter et al. using the Drew–Anderson technique (19). Therefore, the question arises – what will it take to make continuous protective pulmonary ventilation and pulmonary perfusion on bypass a reality in cardiac surgery? We believe that patients at the highest risk for postoperative pulmonary complications and ventilator dependency would benefit the most from a lungs-centered approach, conceptually comparable to the perfusion-supported beating-heart technique in high-risk heart failure patients (18, 20). The time has come to put the vulnerable target organs into primary focus.

## Author Contributions

EG, AG, and TS contributed to the manuscript and reviewed the manuscript.

## Conflict of Interest Statement

The authors declare that the research was conducted in the absence of any commercial or financial relationships that could be construed as a potential conflict of interest.
